# Staring secondaries, where is the primary?

**DOI:** 10.4103/0971-5851.76202

**Published:** 2010

**Authors:** P. Shanmuga Sundaram, S. Padma, Jay Kumar Rai, Vijay Harish

**Affiliations:** *Department of Nuclear Medicine and PET CT, Amrita Institute of Medical Sciences, Cochin, Kerala, India*

**Keywords:** *Metastases*, *metastatic rhabdomyosarcoma*, *positron emission tomography-computed tomography imaging*, *unknown primary*, *uterine tumor*

## Abstract

An asymptomatic issueless young staff nurse underwent pre-employment health screening and USG abdomen showed multiple hypodense lesions in liver. Further screening with whole body positron emission tomography-computed tomography (PET-CT) scan showed significantly FDG avid mass involving most of the right lobe of liver with multiple large FDG avid lymph nodal metastases. Unsuspected focal abnormal, FDG avid, hyperdense mural nodule was seen in uterus, which is the site of primary.

## INTRODUCTION

With the advent of sophisticated medical instrumentation in the diagnostic workup for malignancies, detailed investigations fail to reveal the primary site of origin for a subset of patients with metastatic carcinoma. This is often referred to as Carcinoma of Unknown Primary (CUP) or occult primary malignancy.

The exact incidence of CUP in the United States is not precisely known, but it is definitely underreported. Its actual incidence is most probably between 2 and 6%.[1] In 15–25% of cases, the primary site cannot be identified even on postmortem examination. Identification of a primary site of malignancy poses many challenges and is very important because it dictates the treatment, expected outcome, and overall prognosis.

Clinical presentation of cancer of unknown primary origin is extremely variable and depends on the extent and type of organ involvement. Investigations are usually guided by any positive findings on initial evaluation. Patients have early dissemination of their cancer without symptoms at the primary site. The symptoms often depend on the site of metastases, like ascites may be the initial presentation in a patient with a GI or an ovarian malignancy, etc.

## CASE REPORT

The patient was an issueless, 32-year-old staff nurse undergoing pre-employment health checkup. USG abdomen showed a large heterogenous lobulated mass with predominant hypoechogenicity suggestive of possible atypical hemangioma or possible evolving abscess. Magnetic resonance imaging (MRI) of abdomen showed a large heterogenous enhancing hypodense lesion with altered signal intensity with a large exophytic component in the right lobe of liver suggestive of possible hepatic adenoma/atypical hemangioma/possible neoplasm. Fine needle aspiration cytology (FNAC) followed by trucut biopsy was suggestive of possible metastatic rhabdomyosarcoma.

**Figure 1:**
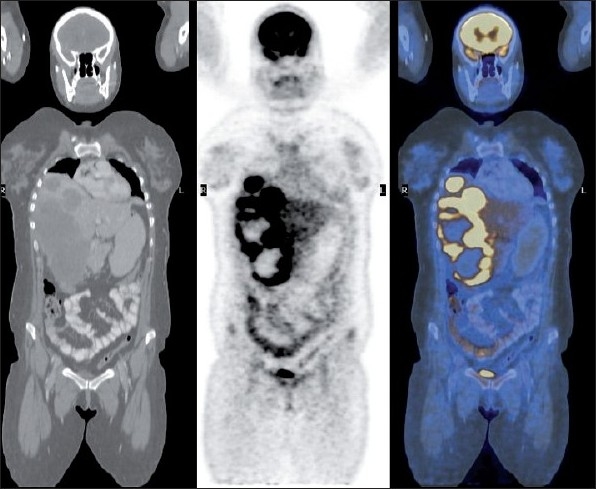
Coronal view of whole body CT, PET and fused images showing large FDG avid liver lesions with multiple necrotic areas. Celiac lymph node deposit also visualized

**Figure 2:**
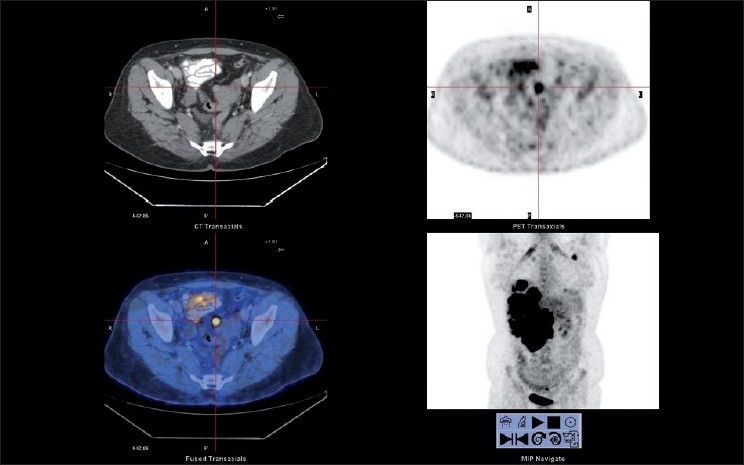
Transaxial view of CT, PET and fused images showing focal FDG avid uterine lesion

Whole body positron emission tomography-computed tomography (PET-CT) scan was done using 8 mCi on ^18^F FDG (Fluoro Deoxyglucose) intravenously in euglycemic status. After 1 hour, the patient was imaged. Images showed large hypodense, significantly FDG avid, non-enhancing mass involving most of the right lobe of liver [Standard Uptake Value (SUV) max 30 g/ml]. Multiple large FDG avid, left axillary, porta hepatic, celiac and retropancreatic lymph nodal metastases were also present. Portal vein was seen encased by the tumor at porta hepatis. Inferior venacava was obstructed below the liver and collateralized via azygos and hemiazygous system. [Figure 1]. Inferiorly, this mass was projecting exophytically out of liver. Presence of another focal abnormal FDG avid, hyperdense mural nodule was noted in the uterus, which was suspected for being the site of primary [Figure 2].

The patient underwent further pelvic USG and was biopsy proven. The patient was started on chemotherapy and is doing well for the past 6 months. Follow-up CT of abdomen showed recession in size of liver metastases.

## DISCUSSION

Rhabdomyosarcoma is primarily a disease affecting children; it rarely affects adults. Common sites of involvement are the head and neck (28%), extremities (24%), and genitourinary tract (18%).[2,3] Uterine rhabdomyosarcoma is rare with only around 60 reported cases, none of them presented with associated liver secondaries. Although most sarcomas are evident on physical examination, it is estimated that 4% of rhabdomyosarcomas and 3–5% of all metastatic cancers present with an unknown primary site.[3] The imaging evaluation of such patients is focused on identifying the primary site. Traditionally, this requires multiple imaging techniques for assessing various anatomic locations. PET-CT allows evaluation of the entire patient in one sitting as a one stop shop.[4]

Most uterine sarcomas fall into the category of leiomyosarcoma, endometrial stromal sarcoma, or undifferentiated sarcoma.[4,5] Metastases are found predominantly in the lungs, bone marrow, bones, lymph nodes, breasts, and brain. Immunohistochemical analysis of tumor cells provides definitive diagnosis.

Rhabdomyosarcoma is subdivided into three general types histologically as follows.

1. Embryonal rhabdomyosarcoma: This usually occurs in head and neck locations with small round or oval tumor cells and a finely granular eosinophilic cytoplasm. Well-differentiated tumors demonstrate elongated, strap-shaped or tadpole-shaped rhabdomyoblasts.
2. Alveolar rhabdomyosarcoma is the next variety comprising relatively small, poorly differentiated round and oval cells aggregated into irregular clusters or nests separated by fibrous septa. An occasional variant, referred to as the botryoid type, shows a diffuse myxoid or mucoid matrix with thinly scattered primitive mesenchymal cells. The characteristic feature of this type is a peripheral zone of increased cellularity, sometimes known as the “cambium layer.”
3. Pleomorphic rhabdomyosarcoma shows randomly arranged eosinophilic cells with considerable variation in cell size and shape, as well as variation in nuclear size and shape. The pleomorphic cells are often admixed with small, primitive mesenchymal cells. This tumor is often so undifferentiated that the identification of the cell of origin is difficult or impossible. Positive immunostains for desmin and myoglobin are helpful as in our case.

Regardless of the histologic subtype, special stains are often quite useful for differentiating rhabdomyosarcoma from other neoplasms. The trichrome stain is especially useful because it colors rhabdomyoblasts bright red while myofilaments and cross-striations have fuchsinophilic properties, also highlighted by PTAH (deep purple color). Myxoid stroma may be positive for hyaluronidase with acid mucopolysaccharide staining, although many other tumors also have positive stroma with these stains.[4] The most useful immunoreactions are toward myoglobin and anti-skeletal muscle actin.[4,5]

In patients with metastatic disease, prognosis is poor with a 5-year event-free survival rate of less than 30%.[6] ^18^F FDG PET-CT is an important screening tool for the evaluation of unknown primaries as well as for staging and follow-up of several malignancies.[7,8] FDG PET imaging capitalizes on the fact that tumors that are highly metabolically active and accumulate more glucose (i.e., FDG) than normal tissue are detected as abnormal. Apart from the visual assessment of images, PET scan also provides an unique quantitative index called Standard Uptake Value (SUV) which is an important yardstick used in the assessment of treatment response in cancer patients.[8]

## CONCLUSION

Our patient was unique, as she was a young asymptomatic lady presenting with multiple large liver and nodal secondaries, which is relatively an uncommon site for metastases. The patient had no previous history of menorrhagia or metrorrhagia as usually expected in uterine rhabdomyosarcomas. Incidentally detected mural nodule in uterus helped in finding the site of hidden primary malignancy.
